# *In-vitro* Antimicrobial Activities of Some Iranian Conifers

**Published:** 2013

**Authors:** Maryam Afsharzadeh, Mahboobe Naderinasab, Zahra Tayarani Najaran, Mohammad Barzin, Seyed Ahmad Emami

**Affiliations:** a *Department of Pharmacodynamic and Toxicology, Faculty of Pharmacy, Mashhad University of Medical Sciences, Mashhad, Iran.*; b *Department of Microbiology, Faculty of Medicine, Mashhad, University of Medical Science, Mashhad Iran.*; c *Department of Pharmacognosy, Faculty of Pharmacy, Mashhad, University of Medical Science, Mashhad, Iran *

**Keywords:** Antimicrobial activity, Cupressus, Cupressaceae, Juniperus, Platycladus, Taxaceae, *Taxus*

## Abstract

Male and female leaves and fruits of eleven different taxons of Iranian conifers (*Cupressus sempervirens *var. *horizontalis*, *C. sempervirens *var. *sempervirens*, *C. sempervirens *cv. Cereifeormis, *Juniperus communis *subsp. *hemisphaerica*, *J. excelsa *subsp. *excelsa*, *J. excelsa *subsp. *polycarpos*, *J. foetidissima*, *J. oblonga*, *J. sabina*, *Platycladus orientalis *and *Taxus baccata*) were collected from different localities of Iran, dried and extracted with methanol. The extracts were tested for their antimicrobial activity against *Pseudomonas aeruginosa*, *Staphylococcus aureus*, *Escherichia coli *and *Candida albicans*. The extracts were screened qualitatively using four different methods, the disc diffusion, hole plate, cylinder agar diffusion and agar dilution methods, whereas the minimum inhibitory concentrations (MIC) of each extract were determined by the agar dilution method. The best result was obtained by means of hole plate method in qualitative determination of antimicrobial activities of extracts and the greatest activity was found against *S. aureus *in all tested methods.

## Introduction

Iranian conifers consist of two families: Cupressaceae and Taxaceae. Cupressaceae consists of one species of *Cupressus *and one species of *Platycladus*. The Taxaceae consists of only one species of *Taxus***. **

*C. sempervirens, P. orientalisand J. excelsasubspexcelsaare *monoecious and others are diecious (1-3). 

An antioxidant activity of methanol extracts of Iranian conifers were investigated previously (4). Cytotoxic study on these plants showed their anti-proliferative activity in cell lines (5-8). 

The progressive resistance of human pathogen microbes against antibiotics, such as methicillin-resistant *Staphylococcus aureus *(MRSA), penicillin-resistant *Streptococcus pneumoniae *(PRSP) and vancomycin-resistant Enterococci (VRE), is a growing problem and it is therefore extremely important to find out and develop new antimicrobial compounds. The screening of plant extracts for their antimicrobial activity has shown that higher plants represent a potential source of new anti-infective compounds (9). 

The antimicrobial efficacy of obtained oils from different species of Iranian conifers was showed previously (10-14).

The purpose of this study is to investigate potential antimicrobial activity of methanol extracts of Iranian conifers, by means of the hole plate, cylinder and disc agar diffusion and agar dilution methods in order to compare the suitability of the screening methods. Whereas the minimum inhibitory concentrations (MIC) of each extract were determined by the agar dilution method.

## Experimental

*Plant material*

Plant specimens were collected from different parts of the country (Table 1). The plants were identified by Dr. M. Assadi, Research Institute of Forest and Rangelands, Ministry of Jahad Keshavarzi, Iran, Voucher specimens of the taxa have been deposited in the Herbarium of National Botanical Garden of Iran (TARI).

**Table 1:** Plant specimens from different parts of the country

**Plant**	**Region**	**Height**	**Date**	**Voucher specimen No**
*C. semipervirens *L. var*. horizontalis *(Mill.) Aiton [syn. *C. horizontalis *Mill.]	Sorkesh, Aliabad Katool, Golestan province	950 m	2 Oct. 2002	72898
*C. semipervirens L. var semipervirens * *[syn. C. Pyramidalis Targ.-Tozz]*	Ecological Garden of Nowshar, Mazandaran province	23 m	5 Oct. 2002	72890
*C. semipervirens *L. cv. Cereifeormis	campus of Ferdowsi University, Mashhad, Khorasan Razavi province	920 m	3 March 2003	72893
*J*. *communis *L. subsp. *hemisphaerica *(Presl) Nyman [syn. *J*. *hemisphaerica *Presl]	between Damulo and Cephali, Golestan province	2063 m	4 Oct. 2002	72897
*J. oblonga *M. Beib.	between Makidi and Vainagh, Arasbaran, East Azarbaijan province	1500 m	6 July 2002	72891
*J. excelsa *M. Beib. subsp *excelsa*	Kelisa Kharabeh, margin of Aras river, East Azarbayejan province	1400-1600 m	30 Nov. 2002	72895
*J. excelsa *M.Beib. subsp. *polycarpos *(K.Koch) Takhtajan [syn. *J. polycarpos *C. Koch]	Chopoughlou Darahsi, East Azarbaijan province	1593 m	21 Sept. 2002	72900
*J. foetidissima *Willd.	Makidi and Vainagh, Arasbaran, East Azarbaijan province	1400 m	23 Sept. 2002	72896
*J. sabina *L.	Sourkesh, Aliabad Katool, Golestan province	2050 m	3 Oct. 2002	No: 72899
*P*. *orientalis *(L.) Franco [syn: *T. orientalis *L.]	Sourkesh, Aliabad Katool, Golestan province	2050 m	2 Oct. 2002	72894
*T. baccata *L.	Armaniolan, Arasbaran, East Azarbayejan province	1175 m	23 Sept. 2002	72892

 The collected materials were stored at -20°C in order to avoid unfavorable changes in the chemical components (15).

*Extraction of the samples*

 Individual fresh leaves of male and female of each plant as well as fruits of them (100 g fresh wt.) were ground by a blinder. Each sample was macerated in pure methanol for 24 h. The samples were then extracted using a percolator. The extracted solutions (27 samples) were concentrated to dryness at 50°C under reduced pressure. The methanol extracts of leaves and fruits of each taxon were evaluated for their antimicrobial activity.

*Isolation and Quantification of alkaloids, flavonoids, saponins and tannins*

 The fruits and leaves of each plant (500 g) were dried at 50°C and then powdered separately. Each powder was defatted with petroleum ether (bp 40-60°C) using Soxhlet apparatus (6 h). The chemical components of defatted powders were extracted by maceration with methanol (four times). The methanol extracts were concentrated at reduced pressure and the presence of alkaloids (16), flavonoids (17), saponins (18) and tannins (19) were determined (Table 2).

**Table 2:** Major components of fruits and leaves of Iranian conifers

**Plant name**	**Plant part**	**Chemical components (average content)** a				
**Alkaloids**	**Flavonoids**	**Saponin**	**Tannins**
*C. semipervirens *var. *horizontalis*	leaves		–	2+	4+	3+
*C. semipervirens *var. *horizontalis*	fruits		–	1+	4+	4+
*C. semipervirens *var. *semipervirens*	leaves		–	1+	1+	1+
*C. semipervirens *var. *semipervirens*	fruits		–	–	1+	2+
*C. semipervirens *cv. Cereifeormis	leaves		–	3+	1+	3+
*C. semipervirens *cv. Cereifeormis	fruits		–	2+	1+	4+
*J. communis *subsp. *hemisphaerica*	leaves (male)		–	3+	1+	4+
*J. communis *subsp. *hemisphaerica*	leaves (female)		–	3+	1+	4+
*J. communis *subsp. *hemisphaerica*	fruits		–	1+	4+	1+
*J. excelsa *subsp. *excelsa*	leaves		–	1+	2+
*J. excelsa *subsp. *excelsa*	fruits		–	2+	1+	1+
*J. excelsa *subsp. *polycarpos*	leaves (male)		–	3+	4+	4+
*J. excelsa *subsp. *polycarpos*	leaves (female)		–	3+	1+	2+
*J. excelsa *subsp. *polycarpos*	fruits		–	2+	1+	2+
*J. oblonga*	leaves (male)		–	3+	1+	4+
*J. oblonga*	leaves (female)		–	3+	1+	4+
*J. oblonga*	fruits		–	3+	1+	2+
*J. foetidissima*	leaves (male)		–	4+	–	3+
*J. foetidissima*	leaves (female)		–	4+	–	2+
*J. foetidissima*	fruits		–	4+	–	2+
*J. sabina*	leaves (male)		–	3+	–	2+
*J. sabina*	leaves (female)		–	3+	–	1+
*J. sabina*	fruits		–	–	–	–
*P. orientalis*	leaves		–	3+	1+	2+
*P. orientalis*	fruits		–	2+	–	3+
*T. baccata*	leaves (male)		2+	4+	4+	3+
*T. baccata*	leaves (female)		3+	2+	–	3+
*T. baccata*	fruits		1+	–	–	4+

a: Average content was rated from - to 4+; +: slightly positive; ++: moderately positive; +++: strongly positive; ++++: very strongly positive; -: not detected.

*Test organisms*

The following microbial strains for testing purposes were purchased from the Persian Type Culture Collection (PTCC): *Escherichia coli *PTCC 1330, *Staphylococcus aureus *PTCC 1337, *Pseudomonas aeruginosa *PTCC 1074, and *Candida albicans *PTCC 5027.

The negative and positive controls were respectively methanol and antibiotics containing discs (gentamycin 10 μg/disc and clotrimazole 8 μg/disc). 

*Hole diffusion method*

This assay was performed using a suspension with 0.5 McFarland standard turbidity. Holes of 6 mm diameter were then made on the Mueller Hinton agar (Merck) plate (8 mm thick) inoculated by flooding and filled with 50 μL of methanol extract. The plates containing the bacteria and *C. albicans *were respectively incubated at 37°C and 25°C for 24 and 48 h. The antimicrobial activity was evaluated by measuring the inhibition zone (IZ) around each hole. They were recorded as (-) for non-active samples and (+) for samples presenting IZ greater than 6 mm (the diameter of the hole) (20).

*Cylinder plate diffusion test*

 This method is the same as hole diffusion test except that the filled cylinder containing 200 μL of different concentration of each extract used as the filled holes. The negative and positive controls were methanol and solutions of two antibiotics (gentamycin 0.2 μg/mL and clotrimazole 0.16 μg/mL), respectively (21).

*Disc diffusion method*

 This assay was performed using the filter paper disc diffusion method on Mueller Hinton and sabouraud dextrose agar respectively for bacteria and *C. albicans*. The plates were incubated under sterile conditions at 37°C for 24 h. Inoculums were prepared using a suspension with 0.5 McFarland standard turbidity and the culture was spread over the plates by means of a sterile cotton swab.

 Plant extracts (0.25-2 mg/disc) were prepared and placed on plates earlier inoculated with microbial suspension. This was done to evaluate the sensitivity of the extracts at which microbial growth was inhibited effectively (22).

*Agar well dilution test*

 Briefly, the methanol extracts (100, 50, 25 and 12.5 μg/mL) were diluted in molten Mueller Hinton agar (MHA, Merck) on 24 well plates. All bacterial strains were grown in Mueller Hinton broth (MHB, Merck) for 4 h at 37°C. Bacterial suspensions with 0.5 McFarland standard turbidity (≈10^8^ cfu/mL), were prepared by dilution with Mueller Hinton broth. The diluted inoculums were added to a Steer’s replicator calibrated and incubated for 24 h at 37°C and 48 h at 25°C respectively for bacteria and *C. albicans *(23).

## Results and Discussion

The antimicrobial activities of 27 methanol extracts of different parts of Iranian conifers were determined using four different methods. Screening was carried out at four different concentrations against *S. aureus*, *E. coli*, *P. aeruginosa *and *C. albicans *strains to examine the sensitivity against the mentioned micro-organism. Table 3 shows the most sensitive microbial strains in different methods.

**Table 3:** Most sensitive microbial strains in different methods

**Method**	***C. albicans***	***E. coli***	***P. aeruginosa***	***S. aureus***
Disc diffusion	_____	_____	*J. excelsa *subsp**. e***xcelsa *leaf	*J. foetidissima *male leaf
Hole plate difffusion	*J. excelsa *subsp**. e***xcelsa *fruit	*T. baccata *male leaf	*C. sempervirens. *cv. Ceriformis leaf	*C. sempervirens*. cv. Ceriformis leaf
	_____	_____	_____	_____
			*C. sempervirens*. cv. Ceriformis leaf	
			*C. sempervirens*. cv. Ceriform fruit	
			*J. excelsa *subsp. e*xcelsa *leaf	
			*J. excelsa *subsp. *polycarpos *female leaf	
			*J. excelsa *subsp. *polycarpos *male leaf	*C. horizentalis *leaf
	*J. excelsa *subsp**. e***xcelsa *leaf		*J. excelsa *subsp. *foetidissima *female leaf	*J. communis*. subsp. *hemisphaerica *female leaf
	*J. polycarpos *male leaf		*J. excelsa *subsp. *foetidissima *male leaf	*J. communis*. subsp. *hemisphaerica *fruit
Cylinder agar dilution	*C. sempervirens. *cv. Ceriformis leaf	*T. baccata *female leaf	*J. excelsa *subsp. *polycarpos *male leaf	*J. excelsa *subsp. *excelsa *leaf
			*J. sabina *female leaf	*J. excelsa *subsp. *polycarpos *female leaf
			*J. sabina *male leaf	*J. excelsa *subsp. *polycarpos *male leaf
			*J. sabina *fruit	*J. oblonga *male leaf
			*T. baccata *female leaf	*P. orientalis *leaf
			*T. baccata *male leaf	

*Determination of micro-organism sensitivity in diffusion methods*

Table 4 shows the antimicrobial activity of Iranian connifer extracts (25, 50, 100 and 200 mg/mL) in three different methods. Methanol extracts exhibited only weak or no activity in cylinder agar diffusion method perhaps because of the low extracts diffusion in agar or may be the precipitation of insoluble material that inhibits further diffusion of active constituent.

**Table 4:** Antimicrobial activity of Iranian connifer extracts for concentration of 25, 50, 100 and 200 g L^-1^ in three different methods.

**Inhibition zone diameter (mm)**											Code/ plant extract		
***S. aureus***			***P. aeruginosa***			***E .coli***			***C. albicans***		***S. aureus***		
**CAD**	**HDP**	**DDM**	**CAD**	**HDP**	**DDM**	**CAD** ^d^	**HDP** ^c^	**DDM** ^b^	**CAD**	**HDP**	**DDM**	**Con.** ^a^	
(-)	11.2±0.26	7±0.2^f^	(-)	(-)	(-)	(-)	(-)	(-) ^e^	(-)	(-)	(-)	25	**C**_1_
(-)	11.83±0.25	7.2±0.25	(-)	(-)	(-)	(-)	(-)	(-)	(-)	(-)	(-)	50	
(-)	12.13±0.2	8.06±0.3	(-)	(-)	(-)	(-)	(-)	(-)	(-)	6.26±0.1	(-)	100	
(-)	12.3±0.26	9.4±1.59	(-)	(-)	(-)	(-)	(-)	(-)	(-)	6.44±0.1	(-)	200	
20.3±1.77	12.73±0.4	20.5±0.87	18.63±1.6	20.43±0.7	18.76±0.35	20.6±1.41	20.7±0.95	18.63±0.47	33.1 ±2.38	34.2±1.87	29.7±0.47	P^g^	
(-)	9.63 ±0.32	6.4 ±0.1	(-)	(-)	(-)	(-)	(-)	(-)	(-)	(-)	(-)	25	**C**_2_
(-)	10.2 ±0.17	7.1 ±0.23	(-)	(-)	(-)	(-)	(-)	(-)	(-)	(-)	(-)	50	
(-)	10.53 ±0.25	8.16 ±0.28	(-)	(-)	(-)	(-)	(-)	(-)	(-)	(-)	(-)	100	
(-)	11.63 ±0.32	8.26 ±0.25	(-)	(-)	(-)	(-)	(-)	(-)	(-)	(-)	(-)	200	
21.7±1.6	21.53 ±1.06	20.4 ±0.79	19±0.45	21.13 ±0.15	19.63 ±0.35	21.3±0.6	20.3 ±1.12	19.16 ±0.1	34.3±0.98	34.1 ±0.6	31 ±0.69	P	
(-)	11 ±0.2	9.1 ±0.17	(-)	10.26 ±0.25	(-)	(-)	(-)	(-)	(-)	(-)	(-)	25	**C**_3_
(-)	12.3 ±0.26	10.86 ±0.32	(-)	10.9 ±0.15	(-)	(-)	(-)	(-)	(-)	(-)	(-)	50	
(-)	14.36 ±0.32	11.9 ±0.1	(-)	11.36 ±0.15	(-)	(-)	(-)	(-)	(-)	(-)	(-)	100	
(-)	15.6 ±0.17	13.5 ±0.5	(-)	12.06 ±0.11	(-)	(-)	(-)	(-)	(-)	6.16 ±0.1	(-)	200	
21.86±1.3	21.2 ±0.36	20.6 ±0.85	18.9±0.5	20.3 ±1	19.46 ±0.76	21.7±0.65	20.86 ±0.8	19.43 ±0.61	35.1 ±0.98	34.6 ±0.56	28.86 ±0.49	P	
(-)	8.2 ±0.26	(-)	(-)	7.43 ±0.15	(-)	(-)	(-)	(-)	(-)	(-)	(-)	25	**C**_4_
(-)	9.33±0.35	6.86±0.5	(-)	7.83 ±0.49	(-)	(-)	(-)	(-)	(-)	(-)	(-)	50	
(-)	10.16±0.2	8.23±0.32	(-)	9.13 ±0.56	6.8 ±0.4	(-)	(-)	(-)	(-)	(-)	(-)	100	
(-)	10.9±0.45	10.03±0.25	(-)	10.16 ±0.15	8.86 ±0.32	(-)	(-)	(-)	(-)	(-)	(-)	200	
21.6±2.3	21.23±0.92	19.8±0.36	19.46±0.7	20.53 ±0.86	18.66 ±0.6	21.46±0.86	20.76±0.9	18.63±0.32	34.8 ±8.5	28.4 ±1.41	30.43 ±0.94	p	
(-)	12.86±0.2	6.4±0.1	(-)	10.33±0.25	(-)	(-)	(-)	(-)	(-)	(-)	(-)	25	**C**_5_
(-)	13.63±0.51	7.23±0.25	(-)	11.4±0.26	(-)	(-)	(-)	(-)	)-(	(-)	(-)	50	
(-)	14.86±0.73	9.7±0.32	(-)	11.96±0.15	(-)	(-)	(-)	(-)	(-)	6.3±0.1	(-)	100	
(-)	15.83 ±0.49	11.83±0.35	(-)	13.4±0.1	(-)	(-)	(-)	(-)	(-)	6.5±0.1	(-)	200	
22.6±1.9	12.2±0.5	20.16±0.5	21.53±0.6	20.83±0.25	20.1±1.13	21.5±0.45	20.1 ±1.3	19.46±0.7	34.8±0.88	34.73±0.45	30±1.21	P	
(-)	11.16±0.15	7.3±0.2	(-)	7.43±0.15	(-)	(-)	(-)	(-)	(-)	(-)	(-)	25	**C**_6_
(-)	12.36±0.35	8.3±0.1	(-)	8.4±0.26	(-)	(-)	)-(	(-)	(-)	(-)	(-)	50	
(-)	14.3±0.15	9.33±0.2	(-)	9.1±0.1	(-)	(-)	)-(	(-)	(-)	(-)	(-)	100	
(-)	14.3±0.1	11.76±0.25	(-)	10.63±0.15	(-)	(-)	)-(	(-)	(-)	(-)	(-)	200	
22.4±20.2	22.06±0.35	20.2±0.45	19.1±0.9	20.06±0.81	20.5±0.65	20.4 ±0.34	20.5±0.65	19.46±0.93	35.3 ±0.7	33.86±0.25	29.7±1.41	P	
(-)	10.13±0.23	7.26±0.25	(-)	(-)	6.43±0.1	(-)	(-)	(-)	(-)	(-)	(-)	25	**C**_7_
(-)	11.2±0.26	8.03±0.1	(-)	(-)	7	(-)	(-)	(-)	(-)	(-)	(-)	50	
(-)	12.13±0.23	9.03±0.1	(-)	(-)	8.03±0.1	(-)	(-)	(-)	(-)	6.4±0.1	(-)	100	
(-)	13.16±0.15	10.16±0.15	(-)	(-)	10.16±0.15	(-)	(-)	(-)	(-)	7.1±0.15	(-)	200	
21.16±1.79 (-)	21.73±0.55 9.3±0.26	21.16±0.41 6.26±0.15	18.85±2.1 (-)	20.13±0.85 (-)	19.4±0.65 (-)	20.5 ±0.6 (-)	20.63±0.8 (-)	19.06±0.85 (-) e	35 ±1.6 (-)	34.7±0.36 (-)	30.06±0.68 (-)	P 25	**C**_8_
(-)	9.43±0.23	7.1±0.17	(-)	(-)	(-)	(-)	(-)	(-)	(-)	(-)	(-)	50	
(-)	10.16±0.2	7.8±0.34	(-)	(-)	(-)	(-)	(-)	(-)	(-)	6.56±0.2	(-)	100	
(-)	12.4±0.69	9.1±0.1	(-)	(-)	(-)	(-)	(-)	(-)	(-)	6.9±0.15	(-)	200	
19.23±0.51	22.3±0.55	20.36±0.45	19.6±0.45	20.16±0.727	19.16±0.15	19.9 ±0.98	20.06±0.56	19.06±0.98	36.7 ±2.8	34.3±0.41	29.06±0.23	P	
(-)	8.83±0.28	6.2±0.1	(-)	(-)	(-)	(-)	(-)	(-)	(-)	(-)	(-)	25	**C**_9_
(-)	9.56±0.1	6.73±0.25	(-)	(-)	(-)	(-)	(-)	(-)	(-)	(-)	(-)	50	
(-)	10.46±0.15	7.03±0.1	(-)	(-)	(-)	(-)	(-)	(-)	(-)	(-)	(-)	100	
(-)	11.06±0.1	7.16±0.28	(-)	(-)	(-)	(-)	(-)	(-)	(-)	6.93±0.1	(-)	200	
21.36±1.02	21.53±0.66	20.63±0.65	19.2±0.75	19.43±0.25	19.23±1.02	21.43±1.8	20.7±1.21	19.96±0.25	34.7±1.49	34.3±0.68	29.83±0.15	P	
(-)	10.26±0.25	7.3±0.55	(-)	7.76±0.25	7.03±0.1	(-)	(-)	(-)	(-)	(-)	(-)	25	**C**_10_
(-)	11.16±0.28	7.8±034	(-)	8.23±0.23	7.2±0.2	(-)	(-)	(-)	(-)	(-)	(-)	50	
(-)	11.93±0.1	9.1±0.17	(-)	10.03±0.2	8.3±0.57	(-)	(-)	(-)	(-)	(-)	(-)	100	
(-)	13.33±0.28	10.4±0.2	(-)	11.5±0.1	9.7±0.26	(-)	(-)	(-)	(-)	(-)	(-)	200	
21.1±1.015	20.76±0.9	20.6±0.75	19.3±0.3	20.36±0.45	20.1±0.26	19.5±1.56	19.76±0.45	18.76±0.35	34.86±1.06	34.8±0.7	30.5±0.8	P	
(-)	11.3±0.26	(-)	(-)	(-)	(-)	(-)	(-)	(-)	(-)	8.03±0.1	(-)	25	**C**_11_
(-)	12.1±0.1	(-)	(-)	(-)	(-)	(-)	(-)	(-)	(-)	8.8±0.1	(-)	50	
(-)	12.43±0.1	(-)	(-)	(-)	(-)	(-)	(-)	(-)	(-)	9.2±0.25	(-)	100	
(-)	13.1±0.1	(-)	(-)	(-)	(-)	(-)	(-)	(-)	(-)	8.83±0.1	(-)	200	
20.26±1.05	21.6±1.3	20.6±1.13	19.1±0.75	20.8±0.45	19.4±0.87	21.6±1.65	20.5±1.04	20.05±0.3	33.8±1.85	35.1±0.55	29.6±1.42	p	
(-)	12.13±0.15	7.2±0.34	(-)	7.2±0.34	(-)	(-)	(-)	(-)	(-)	(-)	(-)	25	**C**_12_
(-)	12.2±0.17	8.16±0.28	(-)	8.16±0.15	(-)	(-)	(-)	(-)	(-)	(-)	(-)	50	
(-)	12.4±0.36	9.5±0.66	(-)	8.8±0.53	(-)	(-)	(-)	(-)	(-)	6.76±0.25	(-)	100	
(-)	13.8±0.32	10.43±0.51	(-)	10.33±0.3	(-)	(-)	6.5±0.2	(-)	(-)	7.06±0.11	(-)	200	
17.7±1.85	21.6±0.62	20.3±0.65	18.76±2.5	19.93±0.41	18.73±0.49	20.6±0.35	19.7±0.45	19.06±0.65	34.96±1.53	34.4±1.08	30.3±07	P	
(-)	10.46±0.15	8.06±0.1	(-)	8.13±0.23	(-)	(-)	(-)	(-)	(-)	(-)	(-)	25	**C**_13_
(-)	12.9±0.17	8.26±0.25	(-)	10.56±0.15	(-)	(-)	(-)	(-)	(-)	(-)	(-)	50	
(-)	14.3±0.3	11.13±0.9	(-)	12.16±0.32	(-)	(-)	(-)	(-)	(-)	6.83±0.15	(-)	100	
(-)	14.4±0.2	11.43±1.25	(-)	11.56±0.15	6.4±0.1	(-)	6.5±0.2	(-)	(-)	7.2±0.2	(-)	200	
19.76±1.75	21.43±1.05	20.56±0.85	20.8±2.7	21±0.62	19.86±0.6	20.5±2.29	21.3±0.45	19.9±0.26	33.5±0.5	33.6±0.65	30.9±0.26	P	
(-)	10.3±0.26	7	(-)	(-)	(-)	(-)	(-)	(-)	(-)	(-)	(-)	25	**C**_14_ **C**_15_
(-)	11.13±0.1	7.03±0.1	(-)	(-)	(-)	(-)	(-)	(-)	(-)	(-)	(-)	50	
(-)	12.2±0.2	7	(-)	(-)	(-)	(-)	(-)	(-)	(-)	(-)	(-)	100	
(-)	12.7±0.25	7.3±0.265	(-)	(-)	(-)	(-)	(-)	(-)	(-)	6.23±0.15	(-)	200	
25±4.7 (-)	20.73±0.37 7.53±0.1	21.03±0.3 8.1±0.17	19.76±1.34 (-)	20.56±0.77 8.53±0.5	18.66±0.4 (-)	20.03±1.7 (-)	19.9±0.62 (-)	19.2±0.55 (-) e	33.1±3.2 (-)	34.56±0.77 (-)	29.2±0.43 (-)	P 25	
(-)	8.5±0.1	9.1±0.15	(-)	9.1±0.15	(-)	(-)	(-)	(-)	(-)	(-)	(-)	50	
(-)	11.43±0.58	11±0.5	(-)	9.86±0.98	(-)	(-)	6.46 ±0.1	(-)	(-)	(-)	(-)	100	
(-)	12.26±0.25	13.6±0.4	(-)	10.9±01.15	(-)	(-)	6.96±0.1	(-)	(-)	7.03±0.1	(-)	200	
21.23±2.4	21.03±0.85	21.23±0.47	20.5±1.53	19.6±0.55	19.63±1.06	16.63±1.5	20.1±0.85	19.03±0.83	34.6±2.2	34.73±0.81	29.2±0.77	Pg	
(-)	8.33±0.35	9.93±0.1	(-)	8.96±0.15	(-)	(-)	(-)	(-)	(-)	(-)	(-)	25	**C**_16_
(-)	9.5±0.5	11.1±0.15	(-)	10.36±0.25	(-)	(-)	(-)	(-)	(-)	(-)	(-)	50	
(-)	12.26±0.25	12.56±0.5	(-)	10.96±0.25	(-)	(-)	8.6±0.2	(-)	(-)	6.83±0.15	(-)	100	
(-)	13.13±0.15	14.26±0.25	(-)	12.26±0.25	(-)	(-)	8.83±0.28	(-)	(-)	7±0.1	(-)	200	
21.36±1.7	21.13±0.66	20.23±1	19.6±1.79	20.36±0.6	18.93±0.4	19.3±0.7	20.4±1.04	19.1±0.96	31.5±1	35.2±0.56	30±1.08	P	
(-)	10.46±0.15	8.06±0.1	(-)	8.13±0.23	(-)	(-)	(-)	(-)	(-)	(-)	(-)	25	**C**_13_
(-)	12.9±0.17	8.26±0.25	(-)	10.56±0.15	(-)	(-)	(-)	(-)	(-)	(-)	(-)	50	
(-)	14.3±0.3	11.13±0.9	(-)	12.16±0.32	(-)	(-)	(-)	(-)	(-)	6.83±0.15	(-)	100	
(-)	14.4±0.2	11.43±1.25	(-)	11.56±0.15	6.4±0.1	(-)	6.5±0.2	(-)	(-)	7.2±0.2	(-)	200	
19.76±1.75	21.43±1.05	20.56±0.85	20.8±2.7	21±0.62	19.86±0.6	20.5±2.29	21.3±0.45	19.9±0.26	33.5±0.5	33.6±0.65	30.9±0.26	P	
(-)	9.73±0.64	7.1±0.17	(-)	(-)	(-)	(-)	(-)	(-)	(-)	(-)	(-)	25	**C**_17_
(-)	11.3±0.26	7.36±0.15	(-)	(-)	(-)	(-)	(-)	(-)	(-)	(-)	(-)	50	
(-)	13.23±0.2	8.26±0.25	(-)	(-)	(-)	(-)	(-)	(-)	(-)	6.76±0.1	(-)	100	
(-)	14.2±0.34	8.33±0.28	(-)	6.9±0.11	(-)	(-)	(-)	(-)	(-)	7.3±0.1	(-)	200	
24.9±6.8	21.76±1	20.26±0.8	18±1.2	20.6±0.17	20.43±1.06	19.93±3.03	20.1±0.32	19.9±0.45	35.3±2.7	34.4±0.96	30.1±1.17	p	
(-)	10. 1±0.17	7.53±0.1	(-)	(-)	(-)	(-)	(-)	(-)	(-)	(-)	(-)	25	**C**_18_
(-)	10.5±0.1	9.06±0.1	(-)	(-)	(-)	(-)	(-)	(-)	(-)	(-)	(-)	50	
(-)	10.9±0.36	9.43±0.81	(-)	7.1±0.1	(-)	(-)	(-)	(-)	(-)	(-)	(-)	100	
(-)	11.06±0.32	10.66±0.75	(-)	7.36±0.15	(-)	(-)	7.1±0.17	(-)	(-)	6.76±0.25	(-)	200	
22.7±1.4	21.73±0.81	19.66±0.3	18.8±1.1	20.23±0.6	19.2±1.21	17.33±2.01	20.1±0.64	19.63±0.8	33.96±3.8	35.1±0.89	30.76±0.3	P	
(-)	9.03±0.1	8.36±0.32	(-)	7.4±1	(-)	(-)	(-)	(-)	(-)	(-)	(-)	25	**C**_19_
(-) (-)	9.5±0.1	9.3±0.2	(-)	8.2±0.2	(-)	(-)	(-)	(-)	(-)	(-)	(-)	50	
	9.96±0.15	9.8±0.28	(-)	9.03±0.1	(-)	(-)	(-)	(-)	(-)	(-)	(-)	100	
(-)	10.26±0.25	10.26±0.25	(-)	7.9±0.15	(-)	(-)	6.93±0.1	(-)	(-)	(-)	(-)	200	
21.6±0.8	21.4±1.03	20.23±0.9	17.6±2.08	21.06±0.4	19.8±0.62	19.1±1.3	20.53±0.92	20.06±0.25	34.1±3.8	35.6±0.1	29.8±0.15	P	
(-)	8.6±0.2	(-)	(-)	7.06±0.25	(-)	(-)	(-)	(-)	(-)	(-)	(-)	25	**C**_20_ **C**_21_
(-)	9.5±0.3	(-)	(-)	7.46±0.1	(-)	(-)	(-)	(-)	(-)	(-)	(-)	50	
(-)	10.26±0.25	7.03±0.1	(-)	8.03±0.1	(-)	(-)	(-)	(-)	(-)	6.46±0.15	(-)	100	
(-)	10.66±0.15	8	(-)	8.5±0.1	(-)	(-)	6.43±0.1	(-)	(-)	7.03±0.15	(-)	200	
22.4±1.49 (-)	21.4±1.3 8.6±0.65	20.9±0.67 7	16.7±1.97 (-)	20.83±0.2 7.3±0.1	19.46±0.58 (-)	20±1.87 (-)	20.1±0.26 (-)	20.4±0.2 (-) e	33.9±2.9 (-)	33.86±0.5 (-)	30.36±0.56 (-)	P 25	
(-)	9.86±0.41	7.23±0.25	(-)	7.8±0.2	(-)	(-)	(-)	(-)	(-)	(-)	(-)	50	
(-)	10.83±0.28	8.03±0.1	(-)	8.83±0.2	(-)	(-)	6.5±0.15	(-)	(-)	7.2±0.26	(-)	100	
(-)	11.56±0.56	9.13±0.15	(-)	9.76±0.2	(-)	(-)	7.5±0.3	(-)	(-)	7.93±0.1	(-)	200	
19.5±0.8	21.8±0.9	21.13±0.2	18.6±1.75	20.7±0.36	19.33±0.3	19.6 ±0.75	20.7±0.3	18.86±0.8	34.1±1.3	35.26±0.45	29.43±1.11	Pg	
(-)	9.46±0.35	7.16±0.28	(-)	8.1±0.17	(-)	(-)	(-)	(-)	(-)	(-)	(-)	25	**C**_22_
(-)	12.16±0.28	8.06±0.1	(-)	8.4±0.2	(-)	(-)	(-)	(-)	(-)	(-)	(-)	50	
(-)	13.2±0.98	8.24±0.1	(-)	11.23±0.15	(-)	(-)	(-)	(-)	(-)	6.96±0.1	(-)	100	
(-)	14.3±0.26	9.06±0.1	(-)	13.1±0.1	(-)	(-)	6.76±0.2	(-)	(-)	7.43±0.1	(-)	200	
24.2±1.2	22.36±0.25	20.43±0.73	18.23±1.6	20.03±0.87	19.36±0.6	19.5±1.27	19.2±0.25	19.5±0.98	36.3±1.38	43.3±0.94	29.93±1.36	P	
(-)	9.36±0.32	6.36±0.15	(-)	7±0.1	(-)	(-)	(-)	(-)	(-)	(-)	(-)	25	**C**_23_
(-)	12.2±0.26	7.26±0.46	(-)	8.06±0.1	(-)	(-)	(-)	(-)	(-)	(-)	(-)	50	
(-)	12.56±0.37	7.96±0.15	(-)	8.4±0.1	(-)	(-)	(-)	(-)	(-)	6.46± 0.15	(-)	100	
(-)	13.16±0.28	8.26±0.3	(-)	10.6±0.3	(-)	(-)	7.03±0.1	(-)	(-)	7.06±0.11	(-)	200	
20.5±0.8	22.36±0.25	20.16±0.6	19.53±1.71	20.83±0.75	19.06±0.82	18.9±3	19.7±0.79	19.73±0.68	35.6±3.36	34.8±0.26	29.96±0.58	P	
(-)	10.4±0.36	7.1±0.17	(-)	6.46±0.15	(-)	(-)	(-)	(-)	(-)	7±0.1	(-)	25	**C**_24_
(-)	10.5±0.43	7.3±0.3	(-)	7.13±0.11	(-)	(-)	(-)	(-)	(-)	7.7±0.26	(-)	50	
(-)	12.06±0.3	8.13±0.15	(-)	7.5±0.1	(-)	(-)	(-)	(-)	(-)	9.06±0.1	(-)	100	
(-)	12.8±0.52	8.6±0.15	(-)	8.2±0.2	(-)	(-)	6.9±0.17	(-)	(-)	9.43±0.2	(-)	200	
23±2.28	21.46±0.65	20.4±0.3	20.3±3.45	20.33±0.65	19.73±0.85	20.23±1.87	19.53±0.49	20.23±0.41	35.5±3	33.86±0.3	28.6±0.63	p	
(-)	7.96±0.1	7.06±0.1	(-)	(-)	(-)	(-)	(-)	(-)	(-)	6.26±0.1	(-)	25	**C**_25_
(-)	9.3±0.2	8.16±0.15	(-)	(-)	(-)	(-)	(-)	(-)	(-)	6.5±0.1	(-)	50	
(-)	11.9±0.36	8.5±0.1	(-)	(-)	(-)	(-)	(-)	(-)	(-)	7.4±0.4	(-)	100	
(-)	12.96±0.45	9.06±0.1	(-)	(-)	(-)	(-)	(-)	(-)	(-)	8.1 ±0.37	(-)	200	
22.9±1.21	21.5±0.65	20.36±0.65	17.93±2.3	19.83±0.1	20.3±0.25	20.93±1.36	20.4±0.53	19.5±0.63	35.9±6	34.6±0.92	29.6±0.55	P	
(-)	7.26±0.25	7.06	(-)	7.4±0.52	(-)	(-)	7.86±0.32	(-)	(-)	(-)	(-)	25	**C**_26_
(-)	8.56±0.51	8.16±0.1	(-)	8.5±0.55	(-)	(-)	8.5±0.1	(-)	(-)	(-)	(-)	50	
(-)	11.4±0.52	8.5±0.4	(-)	9.6±0.88	(-)	(-)	9.03±0.25	(-)	(-)	(-)	(-)	100	
(-)	11.83±0.28	9.06±0.25	(-)	10.96±1.45	10.26±0.64	(-)	9.2±0.1	(-)	(-)	(-)	(-)	200	
21.1±1.4	20.63±0.25	20.36±0.47	18.96±1.4	20.53±0.4	19.53±0.4	19.5±1.5	20.36±0.92	18.83±0.2	33.3±4.9	34.23±1.3	30.5±0.75	P	
(-)	6.83±0.28	(-)	(-)	7.1±0.43	(-)	(-)	(-)	(-)	(-)	(-)	(-)	25	**C**_27_
(-)	7.5±0.3	(-)	(-)	7.26±0.25	(-)	(-)	6.63±0.1	(-)	(-)	(-)	(-)	50	
(-)	8.16±0.15	(-)	(-)	8.8±0.62	(-)	(-)	7.8±0.8	(-)	(-)	(-)	(-)	100	
(-)	9.5±0.5	7.1±0.1	(-)	11.23±0.1	(-)	(-)	10.26±0.25	(-)	(-)	6.3±0.1	(-)	200	
22.06±0.4	21.7±0.65	20.6±0.45	18.26±2.12	20.5±0.75	18.73±0.7	20.4**±**3.2	20.4**±**0.5	18.56±0.25	33±3.1	35.2±0.4	29.23±1.01	P	

a: Con = Concentration; b = DDM: Disc diffusion method; c : HDP = Hole plate difffusion; d : CAD = Cylinder agar diffusion; e: absence of inhibition zones; f: Inhibition zone ≥ 6 showed the antimicrobial activity; g: positive control; Data are shown as mean ± SD of three independent experiments. C_1_ = *C. semipervirens *var. *horizontalis *leaf, C_2 _= *C. semipervirens *var. *horizontalis *fruit, C_3_ = *C. sempervirens *leaf, C_4_ = *C. sempervirens *fruit, C_5_ = *C. sempervirens. *cv. *Ceriformis *leaf, C_6 _= *C. sempervirens. *cv. *Ceriformis *fruit, C_7 _= *J. communis *subsp. *hemisphaerica *female leaf, C_8_ = *J. communis *subsp. *hemisphaerica *male leaf, C_9_ = *J. communis *subsp. *hemisphaerica *fruit, C_10 _= *J. excelsa *subsp. excels leaf, C_11_ = *J. excelsa *subsp. *excelsa *F, C_12 _= *J. excelsa *subsp. *polycarpos *female leaf, C_13 _= *J. excelsa *subsp. *polycarpos *male leaf, C_14_ = *J. excelsa *subsp. *polycarpos *fruit, C_15 _= *J. foetidissima *female leaf, C_16_ =*J. foetidissima *male leaf, C_17 _= *J. foetidissima *fruit, C_18_ = *J. oblonga *fL, C_19_ = *J. oblonga *male leaf, C_20_ = *J. oblonga *fruit, C_21_ = *J. sabina *female leaf, C_22_ =*J. sabina *male leaf, C_23 _= *J. sabina *fruit, C_24_ = *P. orientalis *leaf, C_25_ = *P. orientalis *fruit, C_26_ = *T. baccata *female leaf, C_27_ = *T. baccata *male leaf

 In other methods, the extracts exhibited significant growth inhibition on *S. aureus*. In hole plate method, a weak antibacterial activity against *E. coli *was assessed whereas it showed complete resistance in disc diffusion method as *C. albicans *did. *P. aeruginosa’*s growth was inhibited 62% and 11.1% in hole plate and disc diffusion methods respectively. The extracts were 11.1% effective on *C. albicans *in hole plate assay.

The micro-organism sensitivity in low (25 μg/mL) and high (200 μg/mL) concentrations of extracts were differing in hole plate method as shown in Table 5.

**Table 5:** Comparison of the micro-organism sensitivity in low (25 μg/mL) and high (200 μg/mL) concentrations in hole plate method

**Extract concentration**	**Sensitivity**
**(25 μg/mL)**	*S. aureus *< *P. aeruginosa *< *C. albicans *< *E. coli*
**(200 μg/mL)**	*S. aureus *< *C. albicans *< *P. aeruginosa *< *E. coli*

*MIC of each extracts as determined by agar diffusion method*

The MIC exhibited by the Iranian conifer extracts against tested bacterial and fungal strains are given in Table 6. Obtained results revealed that the extracts are generally more active on *S. aureus *and *P. aeruginosa *whereas *C. albicans *and *E. coli *had higher MIC values.

**Table 6:** Minimal inhibitory concentration (mg/mL) of the methanol extracts of different parts of Iranian conifer species with agar dilution test.

**Plant extract**	**Used part**	***C. albicans***	***E. coli***	***P. aeruginosa***	***S. aurus***
*C. sempervirens *var*. horizontalis*	leaf	100	50	12.5	0.39
	fruit	50	50	25	0.78
*C. sempervirens *var. *sempervirens*	leaf fruit	50 25	100 50	25 25	1.56 1.56
*C. sempervirens *cv. Cereifeormis	leaf fruit	12.5 50	12.5 50	12.5 12.5	0.78 0.78
*J. communis *subsp*. hemisphaerica*	female leaf male leaf	50 50	50 50	25 25	0.39 0.78
	fruit	50	50	25	0.39
*J. excelsa *subsp. *excelsa*	leaf	12.5	25	12.5	0.39
	fruit	50	100	25	0.78
*J. excelsa *subsp*. polycarpos*	female leaf	25	50	12.5	0.39
	male leaf fruit	12.5 50	25 50	12.5 25	0.39 0.78
*J. foetidissima*	female leaf male leaf	25 25	50 50	12.5 12.5	0.78 0.78
	fruit	25	50	25	0.78
*J. oblonga*	female leaf	50	50	25	0.78
	male leaf	25	50	12.5	0.39
	fruit	50	100	25	3.12
*J. sabina*	female leaf	50	50	12.5	0.78
	male leaf	50	50	12.5	0.78
	fruit	50	50	12.5	0.78
*P. orientalis*	leaf	25	500	25	0.39
	fruit	50	50	25	1.56
*T. baccata*	female leaf	25	6.25	12.5	3.12
	male leaf	50	12.5	12.5	3.12

MIC values in mg/mL of extracts and antibiotics. Gentamicin and clotrimazole used as positive controls

*Antimicrobial effects of the investigated Cupressus species*

All of the three tested *cupressus *taxa had antimicrobial properties against *S. aurus*. They have weak or no activity on other strains and this result, except for *S. aurus, *is in agreement with the literature. Alkofahi *et al. *and Guerin *et al. *(24, 25) reported that extracts of *C. sempervirens*: showed no activity against tested strains. The contradictory about *S. aurus *might be due to the extract concentrations, microbial strains or solvent used in our study. Methanol extracts had the most marked antimicrobial effects of all the tested species in different studies (26). *C. sempervirens *and *C. sempervirens. *cv. Cereiformis have been found to possess antimicrobial activities against *P. aeroginosa *in our study. It is believed that this effect shown by these taxa is due to the limitations discussed above. Essential oil of *C. sempervirens *was shown to be a potent inhibitor of *S. aurus *and *P. aeroginosa, *(27) therefore, the presence of essential oil constituent in extracts can be assumed. Since it is known that the leaves and fruits of *cupressus *species contain Camphen, Quercetin, Catechin, Linalool, Borneol and Sabinen, a part of the antimicrobial activities we investigated might be due to these constituents (28-30).

*Antimicrobial effects of the investigated Juniperus species*

As Dornberger *et al. *(31) had found antimicrobial properties in *J. communis*, and Muhammad *et al. *and Topcu *et al. *(32, 33) found evidence of antibacterial activity from the leaves and fruit of *J. excelsa*, it was not a surprise to find antimicrobial extracts among the *Juniperus *species investigated.

Methanol leaves and fruits extracts of all *Juniperus *species were effective against *S. aureus *by hole plat method. All of the *Juniperus *species leaves extracts were shown to be potent inhibitor of *P. aeroginosa.*

Extracts of *J*. *communis *subsp. *hemisphaerica *proved to be effective against *C. albicans *in high concentration with hole plate and among those, female leave extract revealed the greatest activity. The antibacterial effects against *P. aeroginosa *were only shown in the female leave extract by disc diffusion method.

Methanol fruits extract of *J. excelsa *were effective against *C. albicans *which have been investigated by Topcu *et al. *(33) previously.

The antibacterial activity against *P. aeroginosa*, which have been investigated earlier by H24107, was shown in the leave extract.

Except the fruits extract, the other parts of *J. excelsa *subsp*. polycarpos *were found to be effective against *P. aeroginosa*.

Methanol extracts of fruits and leaves of *J. foetidissima *and fruit and female leave of *J. oblanga *were effective against all of the tested microbes by hole plat method. A methanol extract of *J. foetidissima *and *J. oblanga *was added to the tested *Juniperus *extracts to inhibit the growth of *E. coli*.

*Antimicrobial effects of the investigated Platycladus orientalis*

Like other species, methanol extracts of leaves and fruits of *P. orientalis *were effective against *S. aureus *by hole plat method. The antimicrobial activities of methanol extracts of *P. orientalis *did not differ to a marked extent in hole plate method, and were all inhibitors of all the micro-organisms except for *E. coli*. Our antimicrobial results justify the Dornberger *et al. *findings (31). They were only effective against *S. aureus *in disc diffusion method.

*Antimicrobial effects of the investigated Taxus baccata*

Like what stated previously, methanol extracts of leaves and fruits of *T. baccata *were effective against *S. aureus *by hole plat method. Our finding about methanol extracts of *T. baccata *revealed that all of the microbial strains were sensitive except for *C. albicans. *By disc diffusion method, *S. aureus *was the only micro-organism that showed growth inhibition like that obtained with *P. orientalis*.

*Minimum inhibitory concentration values of the extracts*

Methanol extracts of the all taxa gave lowest MIC-values against *S. aureus *(< 3.12 mg/mL) compared to the other strains which had rather higher MIC-values. The reasons for the high MIC values could be that the extracts are mixtures of a large number of compounds and they might suppress the biological activities of each other, or that the active compound(s) is present in very low concentrations. Furthermore, plant extracts generally contain secondary metabolites like saponins, terpenoids and phenolics in a physiologically inactive glycoside form, and this may explain why some of the extracts did not produce very marked inhibition (34). Other reasons could be the slow diffusion of large molecules into the agar and masking detection of the full antimicrobial potential of the extract. This could be overcome by using the turbidimetric method, but there are several problems associated with this method while studying the bioactivities of crude extracts containing large molecules like tannins (35). One major problem is the formation of precipitation in tannin containing crude extracts, which makes it impossible to use turbidity as a measure of bacterial growth. This can be overcome by using INT (*p*-iodonitrotetrazolium violet) which is reduced to the red colored product formazan indicating bacterial growth (36).

Of the 27 extracts of four conifer species we investigated, the most antimicrobial potent one was a leaf extract in methanol of *C. sempervirens. *cv. Ceriformis which had the lowest MIC in comparison with other extract on four strains. Most of the tested plants showed the antimicrobial activity to some extent. It is possible that these essential oils from coniferous trees can be used as antibacterial and/or antifungal agents in food or other ingredients. The mechanism of antibacterial and antifungal effects of these extracted from coniferous trees needs to be further examined for potential uses.

These results indicate that the essential oils derived from coniferous trees, which have mild antimicrobial properties, can inhibit the growth of Gram-positive and Gram-negative bacteria and fungi.
